# Supplementary figures and images for: Detection of toxoplasmic encephalitis in HIV positive patients in urine with hydrogel nanoparticles
Source: PLoS Negl Trop Dis. 2021 Mar 2;15(3):e0009199. doi: 10.1371/journal.pntd.0009199 (PMC7954332; doi:10.1371/journal.pntd.0009199)

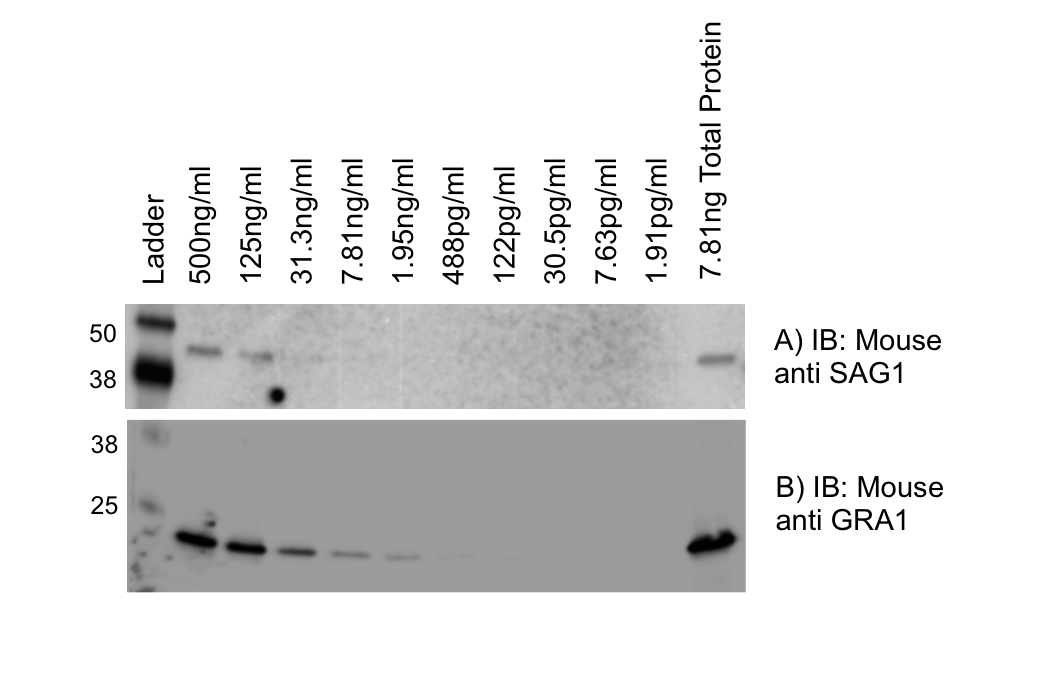

Supplement: S1 Fig — (TIFF) [file pntd.0009199.s003.tiff]
