# Supplementary material for: Detection of toxoplasmic encephalitis in HIV positive patients in urine with hydrogel nanoparticles
Source: PLoS Negl Trop Dis. 2021 Mar 2;15(3):e0009199. doi: 10.1371/journal.pntd.0009199 (PMC7954332; doi:10.1371/journal.pntd.0009199)
Supplement: Attachment — Submitted filename: PLOSNTDs_Response to Reviewers_v3.docx [file pntd.0009199.s004.docx]

Dear Dr. Sinnis and Reviewers;

We thank the reviewers for highlighting the great potential that our urinary test approach holds with respect to the complex diagnostic issues presented by TE. We greatly appreciate your thorough and clear comments on our manuscript “Detection of Toxoplasmic Encephalitis in HIV Positive Patients in Urine with Hydrogel Nanoparticles.” The attention to detail is greatly appreciated. Below you will find your provided comments (in blue) and our specific responses (in black and italicized) to ensure complete response to reviewer concerns. Line numbers are in reference to the track changes version of the manuscript:

**General Editorial Comments**

Specifically it would be important to address the concern that Toxoplasma encephalitis was not definitively diagnosed, bringing into question the significance of this diagnostic test. This can be addressed by limiting the study to patients with evidence of Toxo encephalitis. Further, this method was not compared to a gold-standard. Lastly, reviewers felt that the limitations of the study were not adequately addressed and had additional minor comments that should be addressed.

*We concur that it is unfortunate, for the sake of the study, that there are no participants with the gold standard stereotactic brain biopsy and subsequent histological confirmation of toxoplasmic encephalitis. However, brain biopsy is invasive and carries a nontrivial risk to patients, so is rarely pursued to confirmed suspected TE; additionally, it is not always available in resource-limited settings like our study site. Thus we must consider alternative approaches to diagnosis for the evaluation of our new approach. Quantitative Polymerase chain reaction (qPCR) is accepted as a reference method. qPCR is documented to have near-perfect specificity; however its sensitivity is suboptimal. Thus in the absence of specimens from participants with a gold-standard TE diagnosis, we are able to evaluate the specificity of the nanoparticle western blot capacity and contextualize the sensitivity results against the most commonly available reference diagnostic.*

*Please review the contents of the letter that follows and the new manuscript to address each specific reviewer comment.*

**Specific Reviewer comments**

**Key Review Criteria Required for Acceptance?**
As you describe the new analyses required for acceptance, please consider the following:

**Methods**
-Are the objectives of the study clearly articulated with a clear testable hypothesis stated?
-Is the study design appropriate to address the stated objectives?
-Is the population clearly described and appropriate for the hypothesis being tested?
-Is the sample size sufficient to ensure adequate power to address the hypothesis being tested?
-Were correct statistical analysis used to support conclusions?
-Are there concerns about ethical or regulatory requirements being met?

Reviewer #1: Unfortunately, do not see in their manuscript any info re their patients meeting the minimum criteria for the diagnosis of toxoplasmic encephalitis (TE).

Their patients were HIV infected with neurological symptoms (n= 164), Toxoplasma Ig positive (n= 122, 82%), had low CD4 counts (47-190). However, without additional info such as: brain imaging studies revealing multiple ring enhancing brain lesions, positive response to anti-Toxoplasma Rx, it is quite difficult to assess their true denominator. A suggestion is that the manuscript be revised and resubmitted concentrating only in those patients who had evidence of TE and disseminated toxoplasmosis by CSF and urine PCR, respectively.
*We regrettably only have non-contrast head CTs on 56 study participants. These 56 CTs were reviewed by two radiologists (one Peruvian and one American); however without contrast it was not possible to observe ring enhancement. However, participants had qPCR in CSF and/or blood. We hope we have now clearly differentiated the 41 participants with CSF available for qPCR testing from the remaining 123 participants lacking a CSF specimen, but with a blood specimen (now compared to the urine nanoparticle western blot in supplemental table 1). Inclusion of these 123 participants supplements our knowledge of the applicability of the urine diagnostic and reflects the performance of the assay in clinical applications as requested by the STARD guidelines (https://www.bmj.com/content/bmj/351/bmj.h5527.full.pdf)*. *These participants ultimately enhance the generalizability of the assay through their inclusion of persons from multiple geographical locations. We feel these are important attributes of this assay in this early stage of development.*

For their 164 HIV infected patients with neurological symptoms, there is no statement regarding their consent.
*Please see newly added clarification on patient consent in the manuscript.*

Reviewer #2: Objectives and study are clearly stated.
The study design has limitations, mainly in the definition of patients and definition of patients. However the design remains relevant to test the hypothesis.

Line 147 The population is clearly described, but small and with some biaises (i.e: all controls are from the same hospital). The population is sufficient for a proof of concept but does not allow for a precise assessment of the performances of their test. However, testing controls from other facilities would be an improvement
*We do not have control specimens specifically collected outside of Iquitos. In light of the SARS-CoV-2 global pandemic we are not able to collect more specimens at this time. We do however, have four specimens from Bolivia that were collected from participants were CD4 counts >400 and lacking neurological symptoms. We completed the urine nanoparticle western blot, in accordance with the methods in our manuscript. As you can see all specimens are negative, we however, do not feel this is a sufficient number of specimens to justify their addition to the manuscript.*

Line 190-191 The authors do not specify if the electrotransfert was performed with a tank, semi-dry or dry methods. Please specify

*The transfer occurred in a wet tank, please note the clarification in the methods section of the manuscript. Please refer to line 230*

Line 194 Please confirm if the conjugate is an HRP conjugate

*It is indeed HRP conjugated, please refer to line 234, where this has been clarified*

Line 197-203 The population used to assess performances of the in-house test is too small.

*The Clinical Laboratory Standards Institute (EP09c Measurement Procedure Comparison and Bias Estimation Using Patient Samples; Approved Guideline—Third Edition, Volume 3 Number 11, page 18) requires the testing of 40 specimens for the comparison of a new methodology to a currently approved one in medical laboratories. We acknowledge we fall 2 specimens short of this requirement; however this is minor aspect of the reported results of this paper.*

The performances of the IBL test used in the case definition are, to my knowledge, unpublised.

*IBL Test has been approved by the European Union as an in vitro device for the detection of antibody developed as a result of T. gondii infection. http://www.ibl-international.com/media/catalog/product/R/E/RE57101_IFU_EU_Toxoplasma_gondii_IgG_ELISA_VN-2018-03-13.pdf*

Please add confidence intervals to sensitivity and specificity.

*The confidence intervals have now been included. Please refer to line 243*

This is of minor impact for the study as serology is a very secondary aspect of their work but this should be noted.

Reviewer #3: Are the objectives of the study clearly articulated with a clear testable hypothesis stated? ==> YES
 -Is the study design appropriate to address the stated objectives? ==> Yes. But this study is not going beyond a first basic proof of principle approach. A blinded confirmation study should be designed, or at least mentioned that such validation of the concept is needed

*This suggestion has been incorporated into the final paragraph of the discussion, lines 573-574*
 -Is the population clearly described and appropriate for the hypothesis being tested? ==> as proof of principle, this population is sufficient
 -Is the sample size sufficient to ensure adequate power to address the hypothesis being tested? => as proof of principle, this sample size is sufficient
 -Were correct statistical analysis used to support conclusions? ==> cannot comment
 -Are there concerns about ethical or regulatory requirements being met?  ==> yes

--------------------
 **Results**
-Does the analysis presented match the analysis plan?
-Are the results clearly and completely presented?
-Are the figures (Tables, Images) of sufficient quality for clarity?

Reviewer #1: See comments above

Reviewer #2: Overall: clear results
Line 269-270 Please add standard deviations to median values.

*We have reported the median value because it is a better representation of the data. Standard deviation is a property of the mean. Thus we have instead included a measure of statistical dispersion, i.e. the interquartile range.*

Line 304-305 Figure 2 is blurry and needs higher resolution
Line 323-324 Same for figure 3
*We have removed all low quality images from the manuscript body and included them as separate high quality attachments*

Reviewer #3: Does the analysis presented match the analysis plan?  ==> yes
 -Are the results clearly and completely presented? ==> yes, but there are concerns on the methodology
 -Are the figures (Tables, Images) of sufficient quality for clarity? ==> some Figures need re-evaluation, depending on the revision of the document.

concerns related to the result section:
Line 115, 140, 291:  qPCR: make differentiation between analytical and clinical sensitivity. From the percentages mentioned, it is suggestive to assume this is about clinical sensitivity.

*qPCR has limitations in clinical sensitivity. Nanoparticles increase analytical sensitivity and, in turn, clinical sensitivity. We have disambiguated every instance of the word sensitivity in the text as suggested by the reviewer.*

In line 291: samples are tested twice to increase the sensitivity.  Clarify and add values for both analytical and clinical sensitivity, including Limit of Detection and quantification range. It is not re-assuring if a sample is negative the first time, and after retesting it becomes positive. This can be interpreted as poor assay design and lack of control elements in the experiment. If a sample is negative, and all controls are present, what is the rational for re-testing? 
*We have modified our approach, to only include the results from the first replicate of the qPCR. This change did not affect our qPCR of CSF or blood clot results. Positive controls consist of a pool of positive patient samples and a standard logarithmic curve of RH strain tachyzoites from 1-100000. Samples below 1 tachyzoite were considered negative. (Lines 262-264)*

Line 172:  rewrite sentence

*We have restructured this sentence; we believe this has improved the clarity of our methods. (Lines 208-210)*

Line 298: at what concentration becomes the spiked antigen detectable when not captured with nanoparticles. In absence of a detectable signal in WB, it looks like the experiment as shown in Figure 2 lacks a control for that part of the experiment.

*You will find a western blot exploring the limit of detection of decreasing concentrations of recombinant SAG1 and GRA1 spiked into 1mL of urine as the new first supplemental figure of the manuscript. The limit of detection of SAG1 is 125ng/mL and 1.98ng/mL for GRA1. (Supplemental Figure 1 and lines 344-346)*

The description on Line 178 – 186 does not give the procedure for the control experiment in the approach without nanoparticles.

*This has now been added to the methods section, line 213-216*

Line 319: Figure 3: I am not sure I understand the value of Figure 3A and 3B, and also it is confusing to mention that this is a “representative set” of Peruvian asymptomatic individuals. What is representative on a WB showing only negative results? The value of this Figure 3a and 3b needs more explanation.

*We’ve removed 3A and B from the manuscript; we agree this improves the clarity*

Line 319: Figure 3: Figure 3c and 3d: Is it possible from Figure 2 to quantify the band intensity in Fig3C and 3D. Just in case this is not possible due to the differences in the experimental protocols compared to Figure 2, what is the value of Figure 2? Furthermore, in Fig3D, the bands for - for example SDJ-139 – are also visible in the experiment without nanoparticles. One would assume that the concentration is > 500 pg/ml. Use Figure 2B as a calibrator, the concentration for that sample in Fig 3D (with nanoparticles) is not expected to be higher than 125 pg/ml. The discrepancies in signal intensities needs clarification.

*Unfortunately, it is not possible to use the bands on one gel to quantify those on another due to differences in electro-transfer efficiency and exposure time. These experimental differences are the most likely explanation for the discrepancies in signal intensity between Figures 2 and 3. The low level of GRA1 protein we find in the patients’ samples can further explain these differences.*

Line 321 -323: indication of subsections of Figure 3 needs correction (labelled ad 2A to 2D).

*This has been corrected in the manuscript, lines 352-354*

Line 334: Table 2:  Since qPCR is used as gold standard, it is advisable to have a Limit of detection.

*The limit of detection for the qPCR was a single parasite. (lines 262-263)*

The description of qPCR negative results earlier in the document (line 291) does not give confidence that they are truly negative. Hence the percentages of True Negatives (TN) (97% and 90%) might be biased and influencing Specificity (Line 388).  The False Positives (FP) (3% and 10%) are concerning, and again require clarification on analytical sensitivity of both approaches.
*We have modified our approach, to only include the results from the first replicate of the qPCR. This change did not affect our qPCR of CSF or blood clot results. Thus our data suggests that our initial specificity calculation is correct.*

Line 387: “imperfect gold standard” is difficult to grasp. If a test is not a gold standard, then rather make this a “reference method”. The issue on the gold standard needs a systematic approach, see also Line 437.
*We have corrected this instance and others in the manuscript to refer to qPCR of CSF as the reference standard.*

--------------------

**Conclusions**
-Are the conclusions supported by the data presented?
-Are the limitations of analysis clearly described?
-Do the authors discuss how these data can be helpful to advance our understanding of the topic under study?
-Is public health relevance addressed?

Reviewer #1: See comments above

Reviewer #2: The first part of the conclusion is too strong given the limitations of the study and should be tempered. For instance, line 381 Urine testing could/may provide

*This has been modified in the manuscript, lines 484-485*

Also, the WB test studied is complex and rather long to perform. This should be discussed.

*This point has been added to the limitations section, lines 554-557*

Line 397-398 please add reference to the claim

*Additional citations have been added, line 504*

Outside of the remarks, the conclusion does answer the initial questions and limitations are described and public health relevance is addressed

Reviewer #3: -Are the conclusions supported by the data presented? ==> yes
 -Are the limitations of analysis clearly described? ==> possibly. the value of urine testing is possibly overestimated by lack of proper work on the gold standard.

*This is an excellent point, it has been added to the limitation sections, line 562-563*

Clarification of the analytical sensitivity of the qPCR might give a better understanding of the limitations of the nanoparticle testing. At this moment, the nanoparticle technology concept was attributed a high value because of the limitation on the analytical sensitivity of the gold standard. 

 -Do the authors discuss how these data can be helpful to advance our understanding of the topic under study?  ==> yes.
 -Is public health relevance addressed?  ==> yes

--------------------

**Editorial and Data Presentation Modifications**
Use  this section for editorial suggestions as well as relatively minor modifications of existing data that would enhance clarity. If the only modifications needed are minor and/or editorial, you may wish to recommend “Minor Revision” or “Accept”.

Reviewer #1: N/A

Reviewer #2: The bibliography used as reference is not the best up-to-date in the field but remains relevant.

Reviewer #3: recommendation is:  Minor Revision.

--------------------

**Summary and General Comments**
Use this section to provide overall comments, discuss strengths/weaknesses of the study, novelty, significance, general execution and scholarship. You may also include additional comments for the author, including concerns about dual publication, research ethics, or publication ethics. If requesting major revision, please articulate the new experiments that are needed.

Reviewer #1: Study population included 164 HIV infected patients presenting with neurological symptoms and 51 HIV infected asymptomatic patients from Bolivia and Peru. It is suggested that authors provide the breakdown of their participants by site. The biggest drawback to this study is the absence of "gold standard" for the diagnosis of toxoplasmic encephalitis (TE). Their 122 HIV infected patients who were also positive for T. gondii IgG and had neurological symptoms were at high risk of having TE but do not have more acceptable diagnostic criteria for the TE diagnosis (e.g. multiple brain occupying and ring enhancing lesions, response to anti-Toxoplasma specific Tx). Without these criteria added and well established in their 112 patient population suspected to have TE it is quite difficult to assess the significance of their findings.

Reviewer #2: Overall interesting work with a novel approach for a complex diagnostic. The technique has limitations (long turn around time, performances that still need to defined) but could provide an interesting new option for the specialized labs.

Reviewer #3: The authors should give better attention to the methodology. The urine-based nanoparticle technology has obviously an enormous advantage, but is should be properly compared to a well developed reference method or gold standard. This is missing. It is understandable that the clinical evaluation of the reference method is problematic, but that does not mean that the analytical approach should be excluded. Therefore, the comparison should be at the analytical level, not the clinical sensitivity level. As the results are presented today, this is mixed.

--------------------
