# Supplementary material for: Detection of toxoplasmic encephalitis in HIV positive patients in urine with hydrogel nanoparticles
Source: PLoS Negl Trop Dis. 2021 Mar 2;15(3):e0009199. doi: 10.1371/journal.pntd.0009199 (PMC7954332; doi:10.1371/journal.pntd.0009199)
Supplement: Attachment — Submitted filename: Second Response.docx [file pntd.0009199.s005.docx]

Dear Dr. Sinnis and Reviewer 2;

We again appreciate your comments on our manuscript “Detection of Toxoplasmic Encephalitis in HIV Positive Patients in Urine with Hydrogel Nanoparticles.” Again, below you will find your provided comments (in blue) and our specific responses (in black and italicized) to ensure complete response to reviewer concerns:

Reviewer #2: All concerns previously raised have been answered.
One very minor comment for the authors, with no impact on the study: while the use of CLSI guide might be relevant for validating in-house test it requires to be compared to a validated method and whil IBL is indeed CE-mark I've never heard of it being used in any Toxoplasma reference center in Europe nor seen its evaluation published, thus its performances are unknown.

To your point, we have added a cautionary statement to the manuscript (lines 211-212)

Reviewer #2: One minor comment left. While this comment requires some rewriting of the conclusion, it does not alter the overall findings.
Line 383-385
"CSF PCR is not a gold standard to diagnose TE, thus we could not calculate true clinical sensitivity of the nanoparticle assay. However, CSF PCR is a clinically accepted reference standard because of its specificity. The GRA1 assay exhibited and excellent specificity of 97% compared to CSF PCR."

I disagree. With a 100% specificity on the qPCR, any positive result is a true positive, allowing correct determination of sensitivity (%urine test positive/qPCR positives). However, a negative qPCR can be either a true or a false negative and a positive urine test with negative qPCR might as well be a true or a false positive, hence specificity cannot be calculated.
It would be best to use positive/negative percent agreement, or reinterpret results according to the previous remark (GRA1 has a low sensitivity compared to qPCR and a negative percent agreement of 97%: this could either be due to a sample detected by urine test and not qPCR or a false positive; SAG1 seems of no interest).

Line 393-396 poor sensitivity -> in fact only negatives results when qPCR is positive. Does not change the rest of the section as SAG1 seems indeed of no interest as stated by the authors

We have included positive and negative percent agreement in replacement of specificity in the discussion (lines 390-392 & 402).

We again appreciate all of your comments and efforts to improve the quality of our manuscript. We hope our response adequately addresses all concerns.
